# Prevalence of pain reports in pediatric primary care and association with demographics, body mass index, and exam findings: a cross-sectional study

**DOI:** 10.1186/s12887-018-1335-0

**Published:** 2018-11-21

**Authors:** Randall W. Grout, Rachel Thompson-Fleming, Aaron E. Carroll, Stephen M. Downs

**Affiliations:** 1 Children’s Health Services Research, Department of Pediatrics, Indiana University School of Medicine, 410 W. 10th Street, HS 2000, Indianapolis, IN 46202 USA; 2 Pediatric and Adolescent Comparative Effectiveness Research, Department of Pediatrics, Indiana University School of Medicine, 410 W. 10th Street, HS 2000A, Indianapolis, IN 46202 USA; 3 Regenstrief Institute, Inc, 1101 W. 10th Street, Indianapolis, IN 46202 USA; 4 Present address: Children’s Hospital of Wisconsin, 8915 W Connell Ct, Milwaukee, WI 53326 USA

**Keywords:** Pain, Obesity, Ambulatory care, Clinical decision support

## Abstract

**Background:**

Pediatric pain is associated to patient weight and demographics in specialized settings, but pain prevalence and its associated patient attributes in general pediatric outpatient care are unknown. Our objective was to determine the rate of positive pain screenings in pediatric primary care and evaluate the relationship between reported pain and obesity, demographics, and exam findings during routine pediatric encounters.

**Methods:**

Cross-sectional observational study of 26,180 patients ages 2 to 19 seen in five urban pediatric primary care clinics between 2009 and 2016. Data were collected from systematic screening using a computerized clinical decision support system. Multivariable logistic regressions were used to analyze the association between pain reporting and obesity (body mass index), age, sex, race, season, insurance status, clinic site, prior pain reporting, pain reporting method, and exam findings.

**Results:**

Pain was reported by the patient or caregiver in 14.9% of visits. In adjusted models, pain reporting was associated with obesity (Odds Ratio (OR) 1.23, 95% Confidence Intervals (CI) 1.11–1.35) and severe obesity (OR 1.32, CI 1.17–1.49); adolescents (OR 1.47, CI 1.33–1.61); and females (OR 1.21, CI 1.12–1.29). Pain reported at the preceding visit increased odds of pain reporting 2.67 times (CI 2.42–2.95). Abnormal abdominal, extremity, ear, nose, throat, and lymph node exams were associated with pain reporting. Pain reporting increased in minority races within clinics that predominantly saw a concordant race.

**Conclusions:**

Pain is common in general pediatric encounters, and occurs more frequently in obese children and those who previously reported pain. Pain reporting may be influenced by seasonal variation and clinic factors. Future pediatric pain screening may be guided by associated risk factors to improve identification and targeted healthcare interventions.

## Background

Despite over 16 years of Joint Commission standards to assess and address patient pain, data are sparse regarding the prevalence, demographics, and body metrics associated with pain in general pediatrics. Prior studies have assessed pain in general practice or family practice, but only a fraction of those patients were children. [1, 2] Epidemiological studies in adults describe significant differences in pain prevalence among racial, socioeconomic, sex, and age categories, [3–5] however, comparable studies in pediatrics are sparse. Current scientific knowledge of outpatient pediatric pain is limited to non-routine/emergency [6] or subspecialty/disease-specific [7, 8] settings (e.g., emergency room, obesity clinic, pain clinic), or when pain is the primary complaint/diagnosis. [2, 9] Additionally, epidemiologic studies have focused on chronic pain, [10] especially through surveys at the school, [11] national, [12] or international [13] levels. Among these, general pain estimates ranged between 5% (report of chronic pain at the time of the survey) and 88% (at least one pain episode within the 3 previous months). Site-specific specific pain (e.g., headache, abdominal pain, musculoskeletal pain) rates varied nearly as much, but were also subject to many differences in assessment windows. [10] Few studies assessed the prevalence of pain assessed at the instant of data collection, but these were in school settings. None measured the prevalence of general pain at routine clinical encounters—the largest portion of pediatric healthcare. Even less is known of how clinicians respond to pain reports among children and physical exam findings from visits where pain is reported.

In a 2011 report, the Institute of Medicine requested better data on pain incidence, prevalence, and characteristics, specifically among vulnerable subpopulations, including children, people with low income, and racial and ethnic minorities. [14] This study responds, in part, to this call. Our objective was to determine prevalence of reported pain during a general pediatric encounter, and its relationship with body mass index (BMI) percentile, demographics, socioeconomic status, and season, using records from a clinical decision support system used in pediatric primary care clinics. As secondary outcomes, we investigated physician documentation of pain, and normal versus abnormal physical exam findings associated with reports of pain. Given the existing findings in adults and, to a lesser degree, children, we hypothesized differences in pain exist in pediatric patients based on obesity, sex, age, socioeconomic status, and race, and that pain is associated with an abnormal physical exam and previous pain reports.

## Methods

### Study design and setting

We conducted a cross-sectional observational study using data gathered as part of routine clinical care with the Child Health Improvement through Computer Automation system (CHICA), a clinical decision support system used by five urban, outpatient pediatric clinics in Indianapolis, IN. CHICA was launched in 2004, and has been in continuous use since. CHICA houses data for over 50,000 patients and nearly 350,000 clinical encounters. All clinics are staffed by pediatricians (the majority) or advanced practitioners, referred to as “providers” or “clinicians” herein. These providers receive initial training on CHICA, and have ongoing onsite technical support available. Data and usage patterns are reviewed weekly by the informatics team, and targeted outreach is conducted as needed.

CHICA collects data about patients via two primary means. One is a Pre-Screener Form (PSF), available in English or Spanish, completed by parents or patients in the waiting room. The PSF includes 20 yes/no questions concerning the child’s health and risk factors (e.g., household violence, maternal depression). The questions on the PSF are derived algorithmically [15] based on the child’s age, responses on previous screeners, information from previous clinic visits, and data in the child’s medical record. While initially printed on paper forms, the PSF is now administered on an electronic tablet. [16]

**Fig. 1:**
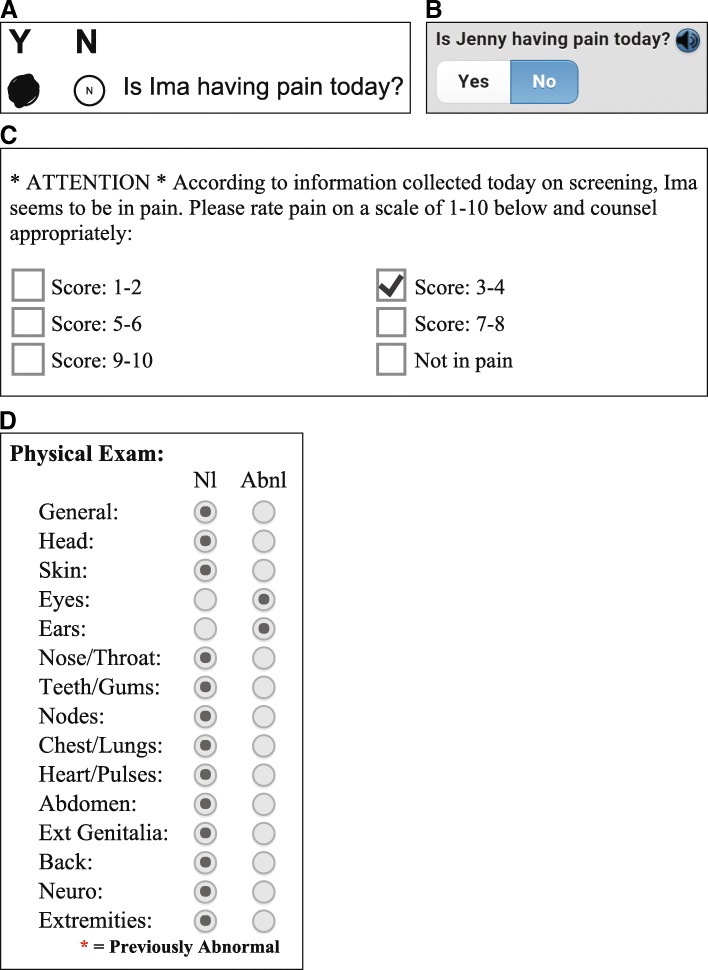
Parts **a**-**d**: User interfaces for CHICA forms. **a** Paper PSF question to assess pain in child. **b** Tablet PSF question to assess pain in child. **c** Positive PSF pain response alert to provider on PWS. **d** Exam documentation template on PWS

The Provider Worksheet (PWS) is the second source of data in CHICA. The PWS contains up to six reminder prompts for treating clinicians that are also algorithmically-generated based on the responses on the child’s PSF(s), data in the electronic medical record, and age-appropriate care guidelines. Each of the six reminders on the PWS contains checkboxes, with which the providers indicate their responses to the prompt. If the patient indicated pain on the PSF screening, the PWS would prompt the provider to document the level of pain and counsel appropriately (Fig. 1c). In addition to decision support, the PWS offers a method to quickly document normal or abnormal exams across 15 body systems, according to each clinician’s assessment (Fig. 1d).

Patients in the clinics using CHICA are diverse, with significant proportions of African American and Hispanic children. CHICA distributes nearly 1000 PSF forms and generates between 2000 and 3000 patient-specific prompts for the various providers on the PWS each month. The overall patient-response rates on the PSF are greater than 85%. CHICA has been used to study a variety of phenomena, such as improved developmental screening [17], infant television viewing and maternal depression [18], and mental health outcomes of children exposed to violence or depression [19].

### Procedure

In this study, we relied on a specific PSF prompt assessing pain that was asked as a routine part of all patient encounters. For patients less than 12 years old, the question was directed to the parent/guardian, reading, “Is [child’s name] having pain today?” (Fig. 1a and b). For patients 12 years and older, with the intention that the child would complete the PSF himself or herself, the question reads, “Are you in pain today?” If the patient reported pain, the physician received the alert in Fig. 1c, with a numeric pain scale reporting option. We recorded the physician responses to the alert and physical exam documented on the PWS at these encounters. We also collected each patient’s pain report from the preceding visit, if available.

We extracted sociodemographic data from CHICA, including race/ethnicity, sex, age, and the payer source at the index encounter. We determined the PSF delivery method—paper versus electronic tablet—based on the date of the visit and the date each clinic transitioned to tablets. Additionally, we classified each visit date by season, using the calendar months as break points (e.g., December through February as winter). We coded age groups based on National Institute of Child Health and Human Development recommendations: 2 to less than 6 years; 6 to less than 12 years; and 12 to less than 20 years of age. We coded race/ethnicity as Black, White, Hispanic, or Other/Unknown. We coded payer source as commercial/private, public (Medicaid/Medicare), and other (self-pay or no insurance).

CHICA records each child’s clinic-measured height and weight. We calculated BMI as weight in kilograms divided by height in meters squared. We determined BMI percentile using Centers for Disease Control and Prevention (CDC) 2000 growth chart parameters. To eliminate biologically implausible values, we discarded data from patient encounters with height, weight, or BMI percentile modified z-score values beyond pre-determined limits by age, following a technique recommended and validated by the CDC. [20]

BMI category was stratified into underweight (less than 5th percentile), normal BMI (5th to less than 85th percentile), overweight (85th to less than 95th percentile), obese (95th to less than 120% of the 95 percentile), and severely obese (120% of the 95th percentile or greater) classes, again following recommendations for stratifying extreme BMI values. [21]

### Data collection

Data were gathered from all patient encounters between July 2009 and February 2016. Eligibility criteria for this study were age between 2 and 19 years, inclusive, and having a PSF pain question response, height, and weight at the same visit. Patient encounters were excluded if any covariate data were missing; no data were imputed. For patients with multiple visits in the CHICA system, we used the last clinical encounter that met eligibility criteria (index encounter).

The study protocol was approved by the Indiana University Institutional Review Board, and included a waiver of informed consent because no interventions were being implemented, impracticability, and minimal risk.

### Statistical analysis

We tested for association between potential variables using Pearson’s chi-squared, with Cramer’s V to measure effect size. Unadjusted (univariable) logistic regression was used to identify variables associated with patient-reported pain, with a threshold of *P* < 0.10 to be retained in an adjusted model. Despite not meeting this cutoff, we retained payer source in the adjusted model as a means-tested proxy for income, and in turn, socioeconomic status (SES). [22] We used multivariable logistic regression models to identify factors associated with positive pain reports. Variables included were BMI category, age category, sex, race, payer source, clinic site, PSF delivery method, season, and pain reported on the PSF at the preceding visit. The most common levels of each variable was chosen as the reference level for the models, except for age, where we chose the youngest group as a chronologic baseline. Due to the association between clinic site and race, we also included an interaction term between clinic and race. We chose to use an interaction fixed effect instead of a random effect for clinic due to the limited number of clinics and to describe the clinic-race mechanisms more deeply. Two models were prepared: the first included all patients at their most recent qualifying visit, and the second was a subset limited to patients for which there was a preceding visit with a response to the pain question. Separately, we created a multivariable logistic regression model to determine the odds ratio of reporting pain based on abnormal exam variables. All multivariable regressions were two-sided tests with alpha = 0.05. We used R 3.3.1 [23] to conduct the analyses.

## Results

**Fig. 2:**
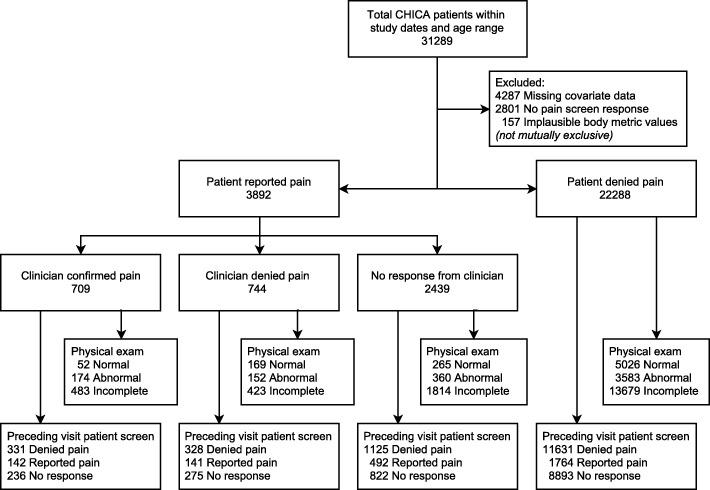
Patient flow and counts through study

Over the study period, 31,289 patients within the appropriate age range were seen; 26,180 met eligibility criteria and their most recent encounter was selected for analysis (see Fig. 2 for patient flow in study).

Table 1 describes patient demographic data. Approximately half of the patients were male (50.8%), and Black patients were the largest race/ethnicity group (51.9%). There were fewer adolescent patients (28.1%) than other age groups. Most visits were covered by public payers (89.2%). Clinics 2 and 3 had more Black patients (87.1 and 82.9%, respectively). Clinic 4 had approximately twice as many Hispanic (45.2%) as Black patients (22.7%). See Table 2 for proportions of clinic populations by race.

**Table 1:** Demographic and patient variables

	Pain reported at index visit		Overall
	No	Yes	
No. with data	22,288	3892	26,180
BMI Class, No. (%)			
Normal BMI	12,793 (57.4)	2037 (52.3)	14,830 (56.6)
Underweight	888 (4.0)	148 (3.8)	1036 (4.0)
Overweight	3636 (16.3)	653 (16.8)	4289 (16.4)
Obese	3233 (14.5)	659 (16.9)	3892 (14.9)
Severely Obese	1738 (7.8)	395 (10.1)	2133 (8.1)
Age, No. (%)			
2–5 years old	7311 (32.8)	1006 (25.8)	8317 (31.8)
6–11 years old	8845 (39.7)	1672 (43.0)	10,517 (40.2)
12–19 years old	6132 (27.5)	1214 (31.2)	7346 (28.1)
Sex = Female, No. (%)	10,808 (48.5)	2064 (53.0)	12,872 (49.2)
Race/ethnicity, No. (%)			
Black	11,711 (52.5)	1878 (48.3)	13,589 (51.9)
Hispanic	6202 (27.8)	1125 (28.9)	7327 (28.0)
White	2145 (9.6)	427 (11.0)	2572 (9.8)
Other/Unknown	2230 (10.0)	462 (11.9)	2692 (10.3)
Insurance type, No. (%)			
Public	19,903 (89.3)	3461 (88.9)	23,364 (89.2)
Commercial	1355 (6.1)	237 (6.1)	1592 (6.1)
Other	1030 (4.6)	194 (5.0)	1224 (4.7)
Clinic site, No. (%)			
Clinic 1	6126 (27.5)	923 (23.7)	7049 (26.9)
Clinic 2	2335 (10.5)	373 (9.6)	2708 (10.3)
Clinic 3	5077 (22.8)	954 (24.5)	6031 (23.0)
Clinic 4	3868 (17.4)	698 (17.9)	4566 (17.4)
Clinic 5	4882 (21.9)	944 (24.3)	5826 (22.3)
Used tablet interface, No. (%)	14,116 (63.3)	2915 (74.9)	17,031 (65.1)
Season, No. (%)			
Autumn	6644 (29.8)	1158 (29.8)	7802 (29.8)
Winter	5299 (23.8)	1131 (29.1)	6430 (24.6)
Spring	3788 (17.0)	736 (18.9)	4524 (17.3)
Summer	6557 (29.4)	867 (22.3)	7424 (28.4)
Pain indicated in preceding visit, No. (%)			
No	11,631 (52.2)	1784 (45.8)	13,415 (51.2)
Yes	1764 (7.9)	775 (19.9)	2539 (9.7)
Unknown (no response)	8893 (39.9)	1333 (34.2)	10,226 (39.1)

**Table 2:** Clinic and race/ethnicity distribution

Variable	Overall (*n* = 26,180)	Clinic 1 (*n* = 7049)	Clinic 2 (*n* = 2708)	Clinic 3 (*n* = 6031)	Clinic 4 (*n* = 4566)	Clinic 5 (*n* = 5826)
Race/ethnicity, No. (%)						
Black	13,589 (51.9)	2526 (35.8)	2359 (87.1)	5000 (82.9)	1037 (22.7)	2667 (45.8)
Hispanic	7327 (28.0)	2764 (39.2)	157 (5.8)	372 (6.2)	2066 (45.2)	1968 (33.8)
White	2572 (9.8)	1034 (14.7)	124 (4.6)	413 (6.8)	435 (9.5)	566 (9.7)
Other	2692 (10.3)	725 (10.3)	68 (2.5)	246 (4.1)	1028 (22.5)	625 (10.7)

The majority of patients had normal BMI percentile (56.6%). The distribution of BMI percentile was skewed left (indicating relatively more frequent obesity); 4% were underweight and 23% obese or severely obese. Approximately 15% of patients reported pain at the index encounter. More than half (15,954 or 60.9%) of all patients had completed pain screening at the preceding visit, and 15.9% of that subset reported pain. There were on average 293 days (SD 269 days) between the index and preceding visits.

All potential variables showed weak or small associations with one another, except for race versus clinic (Cramer’s V = .291, medium correlation). Table 3 presents regression model data. All the variables except payer source were significantly associated with reporting pain in unadjusted models. In the adjusted model, obese and severely obese children had 1.22 and 1.31 times higher odds of reported pain than normal weight children, respectively. Adolescent and middle-childhood patients were each more likely to report pain than younger patients (OR 1.47 and 1.34, respectively). There were higher odds of pain reporting (1) among females, (2) during winter and spring seasons, and (3) when using a tablet interface to record responses. Pain reported at the preceding visit was strongly associated (OR 2.67, CI 2.42–2.95) with pain reports at the index visit.

**Table 3:** Unadjusted and adjusted models predicting a pain report

	Unadjusted Models		Adjusted Model 1^a^ (n = 26,180)		Adjusted Model 2^b^ (*n* = 15,954)	
	OR	90% CI	OR	95% CI	OR	95% CI
BMI Class						
Normal Weight (Ref)						
Underweight	1.05	(0.9–1.21)	1.14	(0.95–1.37)	1.26	(1–1.58)**
Overweight	1.13	(1.04–1.22)*	1.09	(0.99–1.2)	1.12	(0.99–1.27)
Obese	1.28	(1.18–1.39)*	1.23	(1.11–1.35)**	1.2	(1.06–1.36)**
Severely obese	1.43	(1.29–1.58)*	1.32	(1.17–1.49)**	1.26	(1.08–1.48)**
Age						
2–5 years old (Ref)						
6–11 years old	1.37	(1.28–1.47)*	1.33	(1.22–1.45)**	1.13	(1.02–1.26)**
12–19 years old	1.44	(1.33–1.55)*	1.47	(1.33–1.61)**	1.25	(1.11–1.41)**
Sex						
Male (Ref)						
Female	1.2	(1.13–1.27)*	1.21	(1.12–1.29)**	1.2	(1.1–1.3)**
Race/ethnicity						
Black (Ref)						
Hispanic	1.13	(1.06–1.21)*	1.19	(1–1.41)**	1.28	(1.02–1.6)**
White	1.24	(1.13–1.37)*	1.52	(1.23–1.88)**	1.58	(1.18–2.11)**
Other	1.29	(1.18–1.42)*	1.3	(1.02–1.65)**	1.25	(0.89–1.75)
Insurance type						
Public (Ref)						
Commercial	1.01	(0.89–1.13)	1.02	(0.88–1.18)	1.04	(0.86–1.25)
Other	1.08	(0.95–1.23)	1.1	(0.94–1.29)	1.02	(0.81–1.28)
Clinic site						
Clinic 1 (Ref)						
Clinic 2	1.06	(0.95–1.18)	1.13	(0.95–1.35)	1.12	(0.88–1.42)
Clinic 3	1.25	(1.15–1.35)*	1.29	(1.11–1.51)**	1.36	(1.11–1.68)**
Clinic 4	1.2	(1.1–1.31)*	0.75	(0.59–0.96)**	0.98	(0.69–1.39)
Clinic 5	1.28	(1.18–1.39)*	1.01	(0.85–1.21)	1.18	(0.94–1.49)
Pre-screener form (PSF) medium						
Paper (Ref)						
Tablet	1.73	(1.62–1.84)*	1.77	(1.62–1.93)**	1.73	(1.53–1.96)**
Season						
Autumn (Ref)						
Winter	1.22	(1.14–1.32)*	1.21	(1.1–1.32)**	1.2	(1.08–1.35)**
Spring	1.11	(1.02–1.21)*	1.15	(1.04–1.27)**	1.18	(1.04–1.35)**
Summer	0.76	(0.7–0.82)*	0.76	(0.69–0.83)**	0.72	(0.64–0.82)**
Pain reported at preceding visit						
No (Ref)						
Yes	2.86	(2.64–3.11)*			2.67	(2.42–2.95)**
Clinic:Race interaction terms						
Clinic 2:Hispanic			0.71	(0.41–1.17)	0.66	(0.3–1.31)
Clinic 2:White			0.93	(0.54–1.53)	0.86	(0.41–1.7)
Clinic 2:Other			0.55	(0.22–1.19)	0.33	(0.05–1.16)
Clinic 3:Hispanic			0.89	(0.63–1.23)	1.01	(0.66–1.51)
Clinic 3:White			0.78	(0.55–1.09)	0.86	(0.55–1.35)
Clinic 3:Other			0.71	(0.45–1.08)	0.92	(0.51–1.62)
Clinic 4:Hispanic			1.44	(1.08–1.93)**	1.21	(0.81–1.83)
Clinic 4:White			0.87	(0.58–1.29)	0.74	(0.42–1.31)
Clinic 4:Other			1.38	(0.97–1.96)	1.31	(0.8–2.18)
Clinic 5:Hispanic			0.91	(0.72–1.15)	0.83	(0.62–1.12)
Clinic 5:White			0.93	(0.68–1.28)	0.84	(0.57–1.25)
Clinic 5:Other			0.89	(0.64–1.25)	0.85	(0.55–1.32)

^a^Model 1: Adjusted for BMI class, age, sex, race/ethnicity, insurance type, pre-screener form medium, season, with race:clinic interaction terms

^b^Model 2: In addition to Model 1 covariates, includes pain report at prior visit. Limited to patients for whom a pain screen exists at the preceding visit

*P < 0.1, **P < 0.05

There were significant interactions on pain reporting between clinic and race. At the reference site, Black patients had lower odds of reporting pain than other races. Compared to the reference clinic, Black patients at a large clinic that saw primarily (> 80%) Black patients had 29% (CI 1.11–1.51) higher odds of reporting pain. In contrast, Black patients at Clinic 4, a Spanish-English fully bilingual clinic, had lower odds of reporting pain (OR 0.75, CI 0.59–0.96) than at the reference clinic. Within that bilingual clinic, Hispanic patients were 1.72 times (CI 1.36–2.18) more likely to report pain than Black patients.

To confirm this finding, we built a secondary model (not reported in detail here) where the race-clinic interaction was replaced by a variable indicating race-clinic concordance, which was positive when the patient’s race and the predominant race of the clinic were the same. There was a significant 27% increase odds of pain reporting in race-concordant clinics. However, when recreating Adjusted Model 2 from Table 3 (which includes pain reported at the preceding visit), the effect was not significant.

Clinicians documented a scaled pain assessment for 1453 (37%) of the 3892 patients who reported pain, but listed 744 (51%) of those as 0/10 pain (Table 4).

**Table 4:** Clinician pain assessment responses for patients reporting pain

Clinician pain assessment (scale 0–10)	No. (%)
0	744 (19.1)
1–2	321 (8.2)
3–4	187 (4.8)
5–6	114 (2.9)
7–8	52 (1.3)
9–10	35 (0.9)
No response documented	2439 (62.7)

A physical exam for at least one body system was documented through the CHICA PWS on 9781 (37%) of eligible encounters, and a full physical exam (all 15 systems) was documented in 3149 (12%). Table 5 reports descriptive data on abnormal exams for that subset of subjects with a full exam documented.

**Table 5:** Abnormal exams documented in CHICA

	Pain reported at index visit^a^		Overall^a^
	No	Yes	
No. with complete exam documented in CHICA	2838	311	3149
Abnormal Exam component, No. (%)			
Abdomen	87 (3.1)	22 (7.1)	109 (3.5)
Back	43 (1.5)	10 (3.2)	53 (1.7)
Chest/Lungs	73 (2.6)	16 (5.1)	89 (2.8)
Ears/Hearing	98 (3.5)	28 (9.0)	126 (4.0)
External Genitalia	107 (3.8)	18 (5.8)	125 (4.0)
Extremities	87 (3.1)	30 (9.6)	117 (3.7)
Eyes/Vision	97 (3.4)	16 (5.1)	113 (3.6)
General	153 (5.4)	25 (8.0)	178 (5.7)
Head	68 (2.4)	15 (4.8)	83 (2.6)
Heart/Pulses	61 (2.1)	7 (2.3)	68 (2.2)
Neurologic	49 (1.7)	13 (4.2)	62 (2.0)
Nodes	56 (2.0)	20 (6.4)	76 (2.4)
Nose/Throat	190 (6.7)	61 (19.6)	251 (8.0)
Skin	492 (17.3)	73 (23.5)	565 (17.9)
Teeth/Gums	119 (4.2)	12 (3.9)	131 (4.2)

^a^Columns do not sum to total because exam components are not mutually exclusive

In a multiple logistic regression model including only patients with full exams documented, an abnormal exam of the abdomen, ears, extremities, lymph nodes, or nose/throat was associated with higher odds of pain reporting (Table 6). In contrast, heart/pulses and teeth/gum abnormal findings were independently associated with lower odds of pain reporting.

**Table 6:** Multivariable Logistic Regression Model Predicting a Pain Report from Abnormal Exam Components

Abnormal Exam Component	Adjusted OR^a^	95% CI
Abdomen	1.83	(0.99–3.22)*
Back	0.56	(0.18–1.54)
Chest/Lungs	1.30	(0.63–2.47)
Ears/Hearing	2.32	(1.39–3.73)*
External Genitalia	0.93	(0.47–1.7)
Extremities	3.44	(2.03–5.66)*
Eyes/Vision	0.88	(0.45–1.6)
General	1.22	(0.72–1.96)
Head	1.05	(0.5–2.03)
Heart/Pulses	0.22	(0.06–0.69)*
Neurologic	1.50	(0.58–3.46)
Nodes	2.34	(1.21–4.35)*
Nose/Throat	3.15	(2.21–4.44)*
Skin	1.32	(0.98–1.77)
Teeth/Gums	0.31	(0.13–0.64)*

^a^Adjusted model included all terms in this table

*P < 0.05

## Discussion

We examine the prevalence of undifferentiated pain in general pediatric outpatient care. We found about 15% of patients reported pain in our setting, which falls within prior limited clinical and non-clinical estimates among similar age groups or time frames. Previous studies in general or family practice across several countries used pain as the visit reason to estimate prevalence between 5.1 and 36%. [24–26] Within school-based surveys of varying age ranges, point prevalences have been reported between 4.8 and 27.1%. [27–29]

The obesity rates in our study sample were similar to nationally reported statistics. Obesity is linked to several pain outcomes, notably musculoskeletal complaints, headache, and chronic pain. [7, 8] A previous convenience sample showed a positive relationship between BMI and general pain report in an obesity clinic. [30] We show that obesity and severe obesity were associated with higher pain report prevalence in a pediatric population visiting a general outpatient clinic. However, underweight and overweight children did not report more pain. The correlation between obesity and pain may reflect a cycle of decreased exercise due to pain, and pain due to increased BMI from lack of exercise. [31] Unfortunately, many parents do not perceive this association in their own children. [32] Perhaps providers can use this knowledge to incentivize weight reduction to try to reduce pain. However, other psychosocial determinants of health may independently lead to pain and obesity, complicating this relationship.

Our findings are consistent with prior research on characteristics related to pain in children. Females reported pain more often than males, [2, 10] perhaps for psychosociocultural reasons, as the difference is noted across experimental pain response studies, [33] survey instruments, [12] and several pain subtypes. [10, 13] Older children generally report more location specific pain (except for abdominal pain). [10] The lower same-day pain reporting here among younger children may represent a true decrease in pain reports or sensation, a difference between acute and chronic pain among childhood ages, or even a known phenomenon of surrogates (e.g., parents or caregivers) underestimating pain. [34] Insurance at the time of visit was not associated with any change in pain reporting. Past studies demonstrate mixed findings of associations between SES and chronic pain. [10, 35, 36] However, the vast majority of our study population was on public insurance, limiting our power to assess this association. Additionally, the prevalence here includes both chronic and acute pain, and that combination may mask the prior associations with an isolated chronic pain. Increased pain reporting in winter months may reflect known seasonality of common complaints like abdominal pain, headache and respiratory complaints. [37]

In a subset analysis, the strongest predictor of pain reporting was pain reported at the preceding visit. Chronic pain is a notable burden in pediatrics, and it is possible the consecutive pain reports reflect ongoing symptoms. However, it may also be a characteristic of certain patients who are more likely to voice concerns of various common pains.

The largest racial minority in our population reported more pain when in a clinic that predominantly saw patients of that same race. We suspect the social context of patient-clinic racial and cultural concordance may offer a comfort with which to disclose more medical symptoms. Hsieh et al. [38] found increased affective and nonverbal pain expressions when there was both cultural milieu (including language) and race concordance between participants and experimenters – a context similar to the clinics in this population. While it is possible that the race of the treating provider may play a role in pain reporting, we feel this factor is minimized in our data, since the pain question was assessed in the waiting room before interaction with the provider. Further clinical research is needed in facility-level interactions on pain reporting, especially within a new era of computerized clinical decision support systems.

An electronic PSF medium was notably associated with increased pain reports, even when controlling for question response rates. We expected the patient-reported outcomes to be generally equivalent between paper and electronic reporting interfaces. [39] We are uncertain why our experience differs, and it is possible that caregivers and adolescents are more inclined to report pain when the responses go directly to the server instead of on paper, which is handed to clinic personnel. We recommend this topic for future research efforts.

Only 37% of providers rated their patients’ pain after an alert reporting a patient’s positive response. Although the provider response rate to positive pain screens appears low, it is reasonable within the context of historical [40], local [41], and other voluntary clinical reminder system precedent. [42–46] About half (51.2%) of the completed provider assessments confirmed the patient’s pain report, and these were generally rated at low pain levels. It is possible that clinicians were more inclined to respond to the prompt in order to negate what they observed was an erroneous report, which would lead to an artificially depressed rate of providers confirming a patient’s pain report. This perceived error may come from a difference between what the patients are trying to report on the screening question and what providers want to know from the alert. Or, it may simply reflect the disparity between patient, caregiver, and clinician pain assessments. Previous research on concordance of pain assessments by clinicians and patients indicate that providers significantly underestimate pain intensity in both adults and children. [47–49] Pain assessment is just the first step in a pain treatment pathway; one study showed clinicians administered pain relief to fewer than half the patients they determined to be in severe pain. [47] Clinical decision support systems aid in pain screening, but further work is needed enhance provider attention and response. Beyond alerting the clinician, decision support and provider training should focus on pain recognition disparities, and actionable and appropriate treatment recommendations.

Exam findings positively correlated with increased pain may come from common painful conditions within pediatrics, such as acute otitis media, gastroenteritis, extremity injuries, and pharyngitis. The negative correlations may result from routinely documenting an abnormal finding that is not usually painful, like heart murmur or dentition with multiple fillings. It is important to note that our sample of patients with full physical exams is a minority, since clinicians may have documented their exams elsewhere, and it is not routine practice to perform a full physical exam when evaluating a targeted chief complaint. Since our screening question assessed general, undifferentiated pain, it is possible many of the reports were for localized concerns.

The Joint Commission accreditation standard in 2001 described a “patient right” to have pain assessed and addressed. Over the past 16 years, many studies and editorials have discussed the difficulties and outcomes of the universal pain assessment intervention. Authors of these studies/editorials often find little or no effect on pain control attributable to systematic pain assessment, and there is controversy over the timing of an “opioid crisis” and the Joint Commission mandate to assess and address pain. [50] Whether systematic pain assessment improved outcomes for the patients in our study is uncertain, but pain was associated with a subset of physical findings, suggesting that there is diagnostic information in the pain measure.

Our study is not without limitations. First, CHICA PSF questions have binary (yes/no) responses, and clinicians are not required to follow-up with a standardized pain assessment. Since pain reports were generally rated by parents or providers, it is possible our findings underestimate the true pain prevalence in this population. It is possible that clinicians were documenting pain or physical exams independently in their notes, which would not be captured with our clinical decision support system. Our observational, cross-sectional study design is unable to identify causal relationships; for example, physical exam findings may be from other abnormalities that are not painful. We do not have data to differentiate well visits versus sick visits, or acute versus chronic pain, which is likely to affect pain reporting, and we emphasize our results are from routine, systematic screening. At the time of this study, the medical record data did not differentiate between race and ethnicity, and so the effect of these two distinct constructs is blurred. Finally, our data come from five urban pediatric primary care clinics serving predominantly low-income and minority families living in a single metropolitan area, so readers should exercise caution in generalizing these results.

## Conclusions

Using a large diverse sample, we establish the first data on prevalence of pain and associated characteristics in general pediatric primary care. We show that previously reported trends in pain epidemiology in fact extend to the ambulatory practice environment. More than one in seven patients reported pain at these encounters. Our data suggest that females, White children, children 5 years of age or older, and obese or severely obese children had higher odds of reporting pain at the time of a clinical encounter. In addition, Black and Hispanic patients reported more pain in clinics with concordant majority races. Pain reports were also positively associated with electronic reporting interfaces, certain seasons, and specific exam findings. Our study answers an Institute of Medicine request for better data on childhood pain, especially within a minority and low socioeconomic status population, and provides a springboard for research into such a common symptom in primary care. Future studies should apply methods [51] that improve pain assessment workflows and efficiency in general pediatric practice, and assess clinical outcomes associated with pain assessment in children.

## Abbreviations

ADHD: Attention deficit hyperactivity disorder
BMI: Body mass index
CHICA: Child Health improvement through computer automation
PSF: Pre-screener form
PWS: Provider worksheet

## References

[1] Hasselström J, Liu-Palmgren J, Rasjö-Wrååk G (2002). Prevalence of pain in general practice. Eur J Pain.

[2] Knapp DA, Koch H. The management of new pain in office-based ambulatory care: National Ambulatory Medical Care Survey, 1980 and 1981. Adv Data. 1984;97:1–9. https://www.ncbi.nlm.nih.gov/pubmed/10266817. 10266817

[3] Nahin RL (2015). Estimates of pain prevalence and severity in adults: United States, 2012. J Pain Off J Am Pain Soc.

[4] Riskowski JL (2014). Associations of socioeconomic position and pain prevalence in the United States: findings from the National Health and nutrition examination survey. Pain Med.

[5] Campbell CM, Edwards RR (2012). Ethnic differences in pain and pain management. Pain Manag.

[6] Drendel AL, Brousseau DC, Gorelick MH (2006). Pain assessment for pediatric patients in the emergency department. Pediatrics.

[7] Wilson AC, Samuelson B, Palermo TM (2010). Obesity in children and adolescents with chronic pain: associations with pain and activity limitations. Clin J Pain.

[8] Smith SM, Sumar B, Dixon KA (2014). Musculoskeletal pain in overweight and obese children. Int J Obes.

[9] Caudill-Slosberg MA, Schwartz LM, Woloshin S (2004). Office visits and analgesic prescriptions for musculoskeletal pain in US: 1980 vs. 2000. Pain.

[10] King S, Chambers CT, Huguet A, MacNevin RC, McGrath PJ, Parker L (2011). The epidemiology of chronic pain in children and adolescents revisited: a systematic review. Pain.

[11] Roth-Isigkeit A, Thyen U, Stöven H, Schwarzenberger J, Schmucker P (2005). Pain among children and adolescents: restrictions in daily living and triggering factors. Pediatrics.

[12] Perquin CW, Hazebroek-Kampschreur AAJM, Hunfeld JAM, Bohnen AM, van Suijlekom-Smit LWA, Passchier J (2000). Pain in children and adolescents: a common experience. Pain.

[13] Swain MS, Henschke N, Kamper SJ, Gobina I, Ottová-Jordan V, Maher CG (2014). An international survey of pain in adolescents. BMC Public Health.

[14] Institute of Medicine (US) Committee on Advancing Pain Research, Care, and Education. Relieving Pain in America: A Blueprint for Transforming Prevention, Care, Education, and Research. Washington (DC): National Academies Press (US); 2011. http://www.ncbi.nlm.nih.gov/books/NBK91497/. Accessed 16 Apr 2016. 22553896

[15] Anand V, Carroll AE, Biondich PG, Dugan TM, Downs SM. Pediatric decision support using adapted Arden syntax. Artif Intell Med. 2015. 10.1016/j.artmed.2015.09.006. 10.1016/j.artmed.2015.09.006 PMC4818208 26547523

[16] Anand V, McKee S, Dugan TM, Downs SM (2015). Leveraging electronic tablets for general pediatric care: a pilot study. Appl Clin Inform.

[17] Carroll AE, Bauer NS, Dugan TM, Anand V, Saha C, Downs SM (2014). Use of a computerized decision aid for developmental surveillance and screening: a randomized clinical trial. JAMA Pediatr.

[18] Anand V, Downs SM, Bauer NS, Carroll AE (2014). Prevalence of infant television viewing and maternal depression symptoms. J Dev Behav Pediatr JDBP.

[19] Bauer NS, Gilbert AL, Carroll AE, Downs SM (2013). Associations of early exposure to intimate partner violence and parental depression with subsequent mental health outcomes. JAMA Pediatr.

[20] Freedman DS, Lawman HG, Pan L, Skinner AC, Allison DB, McGuire LC (2016). The prevalence and validity of high, biologically implausible values of weight, height, and BMI among 8.8 million children. Obes Silver Spring Md.

[21] Flegal KM, Wei R, Ogden CL, Freedman DS, Johnson CL, Curtin LR (2009). Characterizing extreme values of body mass index–for-age by using the 2000 Centers for Disease Control and Prevention growth charts. Am J Clin Nutr.

[22] Cheng TL, Goodman E, Committee on Pediatric Research (2015). Race, ethnicity, and socioeconomic status in research on child health. Pediatrics.

[23] R Core Team (2018). R: a language and environment for statistical computing.

[24] Zailinawati AH, Teng CL, Kamil MA, Achike FI, Koh CN (2006). Pain morbidity in primary care - preliminary observations from two different primary care settings. Med J Malaysia.

[25] Mäntyselkä P, Kumpusalo E, Ahonen R, Kumpusalo A, Kauhanen J, Viinamäki H (2001). Pain as a reason to visit the doctor: a study in Finnish primary health care. Pain.

[26] Frølund F, Pain in General Practice FC (1986). Pain as a cause of patient-doctor contact. Scand J Prim Health Care.

[27] van Dijk A, McGrath PA, Pickett W, VanDenKerkhof EG. Pain prevalence in nine- to 13-year-old school children. Pain Res Manag J Can Pain Soc 2006;11:234–240. 10.1155/2006/835327 PMC2673140 17149456

[28] Huguet A, Miró J (2008). The severity of chronic pediatric pain: an epidemiological study. J Pain.

[29] Barajas C, Bosch F, Baños J-E (2001). A pilot survey of pain prevalence in schoolchildren. Pain Clin.

[30] Hainsworth KR, Miller LA, Stolzman SC, Fidlin BM, Davies WH, Weisman SJ (2012). Pain as a comorbidity of pediatric obesity. Infant Child Adolesc Nutr.

[31] Rabbitts JA, Holley AL, Karlson CW, Palermo TM (2014). Bidirectional associations between pain and physical activity in adolescents. Clin J Pain.

[32] Hainsworth Keri, Jastrowski Mano Kristen, Stoner Alison, Anderson Khan Kim, Ladwig Renee, Davies W., Defenderfer Ellen, Weisman Steven (2016). “What Does Weight Have to Do with It?” Parent Perceptions of Weight and Pain in a Pediatric Chronic Pain Population. Children.

[33] Boerner KE, Birnie KA, Caes L, Schinkel M, Chambers CT (2014). Sex differences in experimental pain among healthy children: a systematic review and meta-analysis. Pain.

[34] Chambers CT, Reid GJ, Craig KD, PJ MG, Finley GA (1998). Agreement between child and parent reports of pain. Clin J Pain.

[35] Perquin CW, Hazebroek-Kampschreur AA, Hunfeld JA, van Suijlekom-Smit LW, Passchier J, van der Wouden JC (2000). Chronic pain among children and adolescents: physician consultation and medication use. Clin J Pain.

[36] Korterink Judith J., Diederen Kay, Benninga Marc A., Tabbers Merit M. (2015). Epidemiology of Pediatric Functional Abdominal Pain Disorders: A Meta-Analysis. PLOS ONE.

[37] Schrijver TV, Brand PLP, Bekhof J (2016). Seasonal variation of diseases in children: a 6-year prospective cohort study in a general hospital. Eur J Pediatr.

[38] Hsieh AY, Tripp DA, Ji L-J (2011). The influence of ethnic concordance and discordance on verbal reports and nonverbal behaviours of pain. Pain.

[39] Muehlhausen W, Doll H, Quadri N, Fordham B, O’Donohoe P, Dogar N (2015). Equivalence of electronic and paper administration of patient-reported outcome measures: a systematic review and meta-analysis of studies conducted between 2007 and 2013. Health Qual Life Outcomes.

[40] McDonald CJ (1976). Protocol-based computer reminders, the quality of care and the non-perfectability of man. N Engl J Med.

[41] Bauer NS, Carroll AE, Saha C, Downs SM (2016). Experience with decision support system and comfort with topic predict clinicians’ responses to alerts and reminders. J Am Med Inform Assoc.

[42] Zheng K, Padman R, Johnson MP, Diamond HS (2005). Understanding technology adoption in clinical care: clinician adoption behavior of a point-of-care reminder system. Int J Med Inf.

[43] Meigs JB, Cagliero E, Dubey A, Murphy-Sheehy P, Gildesgame C, Chueh H (2003). A controlled trial of web-based diabetes disease management: the MGH diabetes primary care improvement project. Diabetes Care.

[44] van Wyk JT, van Wijk MAM, Sturkenboom MCJM, Mosseveld M, Moorman PW, van der Lei J (2008). Electronic alerts versus on-demand decision support to improve dyslipidemia treatment: a cluster randomized controlled trial. Circulation.

[45] Green LA, Nease D, Klinkman MS (2015). Clinical reminders designed and implemented using cognitive and organizational science principles decrease reminder fatigue. J Am Board Fam Med.

[46] Litzelman DK, Dittus RS, Miller ME, Tierney WM (1993). Requiring physicians to respond to computerized reminders improves their compliance with preventive care protocols. J Gen Intern Med.

[47] Brudvik C, Moutte S-D, Baste V, Morken T (2017). A comparison of pain assessment by physicians, parents and children in an outpatient setting. Emerg Med J.

[48] Singer AJ, Gulla J, Thode HC (2002). Parents and practitioners are poor judges of young children’s pain severity. Acad Emerg Med.

[49] Mäntyselkä P, Kumpusalo E, Ahonen R, Takala J (2001). Patients’ versus general practitioners’ assessments of pain intensity in primary care patients with non-cancer pain. Br J Gen Pr.

[50] Baker DW. Joint Commission Statement on Pain Management. http://www.jointcommission.org/joint_commission_statement_on_pain_management/. Accessed 19 Dec 2017.

[51] Simons LE, Smith A, Ibagon C, Coakley R, Logan DE, Schechter N (2015). Pediatric pain screening tool: rapid identification of risk in youth with pain complaints. Pain.

